# Magnitude of asbestos-related lung cancer mortality in Italy

**DOI:** 10.1038/sj.bjc.6604450

**Published:** 2008-06-24

**Authors:** A Marinaccio, A Scarselli, A Binazzi, M Mastrantonio, P Ferrante, S Iavicoli

**Affiliations:** 1 Epidemiology Unit, Department of Occupational Medicine, Italian National Institute for Occupational Safety and Prevention, Via Alessandria 220/E, Rome 00198, Italy; 2 Unit of Toxicology and Biomedical Sciences, Italian National Agency for New Technologies, Energy and the Environment, Via Anguillarese 301, S.Maria di Galeria, Rome 00060, Italy

**Keywords:** asbestos, mortality, lung cancer, pleural cancer, Italy

## Abstract

An ecological study, based on a data set containing all lung and pleural cancer deaths in each Italian municipality in the period 1980–2001, was performed. The pleural to lung cancer ratio was estimated to be 1 : 1 and 3% (around 700) of all male lung cancer deaths were found to be asbestos-related.

Asbestos is the most relevant occupational carcinogenic agent for the human lung considering its carcinogenic power and the large amount of exposed workers. The epidemiology of asbestos-related lung cancer (LC) cannot be investigated directly because cases are not clinically distinguishable from those due to other causes. The magnitude of the association and the attributable risk have been estimated in cohorts of workers exposed to asbestos and from LC case–control studies (Albin *et al*, 1999), but few studies have investigated it in the general population because generalisation of these findings is problematic (De Vos, 1993; Darnton *et al*, 2006). Ratios ranging from 1 : 1 to 1 : 10 between mesothelioma and excess LC deaths due to asbestos exposure have been reported in different cohorts of exposed workers (Vineis and Simonato, 1991).

An ecological study based on a data set containing all lung and pleural cancer deaths in each Italian municipality in the period 1980–2001 was performed among males. The purpose was to examine the association between LC and pleural cancer mortality (the latter as a proxy of asbestos exposure), and to estimate the proportion of LC deaths attributable to asbestos exposure in the past.

## Methods

The geographic distribution of LC has been analysed among Italian municipalities in terms of demographic (urbanisation degree), geographic (altitude) and socioeconomic (deprivation index) features. The distribution of deaths from pleural tumours has been assumed as a marker of territorial asbestos exposure in the past and used to estimate the magnitude of asbestos-related LCs by a multiple regression model.

Mortality data (source: the Italian National Institute of Statistics) were made available by the Epidemiological Database of the Italian National Agency for New Technologies, Energy and the Environment. According to the Ninth International Classification of Diseases (IX ICD), codes 163.0–163.9 for malignant neoplasm of pleura and 162.0–162.9 for lung tumours were selected. Crude and standardised mortality rates (per 100 000 inhabitants) and ratios, using national age-specific mortality rates to derive expected numbers, were estimated in each Italian municipality (*n*=8101) in the period 1980–2001. The urbanisation degree is a summary index, estimated by the Italian National Institute of Statistics, of 14 demographic and economic aspects. Each municipality has been classified as urban, semiurban, semirural and rural (for a detailed description visit the website http://www.istat.it). Altitude was included in the analysis with three categories (more than 500 m of altitude; between 250 and 500 m and less than 250 m). The deprivation index is a complex municipal parameter of the degree of poverty and is extensively described elsewhere (Cadum *et al*, 1999). For each component, the standardised difference from the national average value was calculated, summarised and finally each municipality was coded as very deprived, deprived, medium, rich and very rich.

A univariate analysis was performed to verify the relationship between LC male mortality among all municipalities, deprivation index, urbanisation, altitude and pleural cancer male mortality. The significance of differences among LC mortality rates was tested by one-way analysis of variance and the correlations with pleural cancer by Pearson's coefficient (*r*).

A multivariate analysis was conducted by way of a set of linear generalised regression models considering the observed deaths due to LC (O) as response variable. Altitude (A), urbanisation level (U) and deprivation index (D) were considered in the models as factors and pleural cancer risk (PL) as covariate. The functional expression for the most comprehensive model is 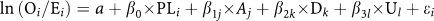 where *i*=1,…*N*=8101 Italian municipalities and *j*=1,…, 3; *k*=1,…,5; *l*=1,…, 4 for the coefficients of explanatory variable. E is the number of expected deaths due to LC, *α* the intercept and *ε* the random error with a normal distribution.

Different models were designed according to the set of explanatory variables included. The goodness of fit (*R*^2^) was assessed for each model tested and the models with the highest values are reported in Table 2. Estimated regression coefficients were calculated and used to compute the number of LC deaths predicted in each municipality; for each model, a second prediction were obtained by setting to zero the coefficient (*β*_0_) of pleural cancer mortality risk variable, assuming no exposure to asbestos in the past. The estimated number of asbestos-related LC deaths is the difference between the two predicted totals. The proportion of these asbestos-related – with respect to the total – LC deaths was calculated and finally the ratio of pleural cancer to asbestos-related LC deaths for each model was estimated.

All statistical analyses were performed using the Statistical Package for Social Sciences (SPSS version 15.0).

## Results

**Figure 1:**
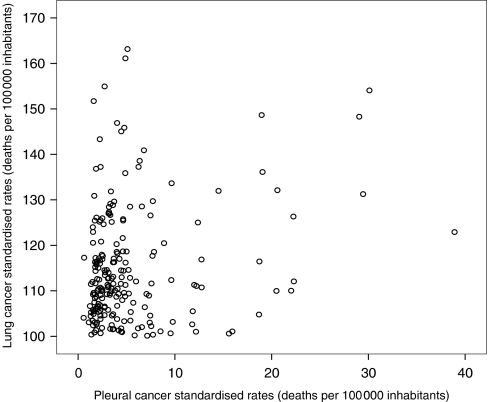
Correlation between pleural cancer and lung cancer standardised mortality rates. All Italian municipalities (*N*=8101). Italy, 1980–2001, males. Pearson's correlation *r*=0.15; *P*<0.001. Municipalities with less than 3 pleural cancer deaths, with less than 20 lung cancer deaths or a standardised lung cancer mortality rate less than 100 (per 100 000 inhabitants) are not shown.

In the Italian male population, 536 538 LCs and 12 216 pleural cancer deaths were recorded in the period 1980–2001, with annual standardised rates of 87.91 and 2.00 (per 100 000), respectively. Among municipalities with at least 10 pleural cancer deaths in the selected period, Casale Monferrato (Piedmont, north-western Italy), Broni (Lombardy, northern Italy) and Monfalcone (Friuli-Venezia Giulia, north-eastern Italy) present the highest rates (38.9, 29.5 and 22.3, respectively), reflecting the historical high occupational and environmental contamination from the asbestos cement industry in the first two municipalities (Magnani *et al*, 1997; Amendola *et al*, 2003) and from shipbuilding and repair in Monfalcone (Bianchi *et al*, 1993). Figure 1 shows a direct significant correlation between LC and pleural cancer standardised mortality rates among all Italian municipalities (Pearson's correlation coefficient *r*=0.15; *P*<0.001). Previous geographic analyses (Mastrantonio *et al*, 2002; Marinaccio *et al*, 2007) demonstrated that the clusters of pleural cancer deaths identify areas involved in intensive asbestos use in the past, justifying its use as a proxy variable of past asbestos exposure.

In Table 1, the annual age standardised rates for LC are reported by altitude, urbanisation, deprivation and pleural cancer mortality, with LC showing a linear trend with altitude (analysis of variance: *P*<0.001) and being significantly less frequent in the 2150 ‘rural’ municipalities. Correlation with deprivation index is less clear but a lower LC rate among ‘very-rich’ municipalities is evident. A significant correlation between LC and pleural cancer rates was found whether the latter was considered continuous or categorised (Figure 1 and Table 1).

Results of the regression analysis are presented in Table 2. The goodness of fit ranges between 0.02 and 0.3, and the estimated asbestos-related LC deaths in Italy amount to 1.6–3.7% of all LC deaths (between 380 and 770 deaths per year). For each mesothelioma death in Italy, we estimated between 0.68 and 1.37 LC deaths. The strong correlation between urbanisation and deprivation measure (*χ*^2^ test: *P*<0.001) suggests the assumption of the models with one of these variables (model 2 or 3) as most reliable, and consideration of model 4, although with the best fit, as not adequate. The comparative evaluation of the models induce to consider a pleural cancer to LC ratio of 1 : 1 and 3% of all LC deaths as asbestos-related as the most reliable predictions, with an average magnitude of around 700 asbestos-related LCs per year among males in Italy.

## Discussion

The extent of asbestos-related LCs at national population level is not often considered in Italy (where it has been banned in 1992), despite its importance as an occupational neoplasm in the context of epidemiology, prevention and compensation in UK. Using a linear model, the total excess LC deaths due to asbestos exposure was estimated at 2–3% of total male LC deaths and its ratio to mesothelioma deaths was between 1 : 1.5 and 1 : 1 (Darnton *et al*, 2006). In Italy, a study of LC mortality in an asbestos cement-manufacturing town (Casale Monferrato) reported an attributable risk among exposed of 67.5% for men and 51.3% for women, and among the general population (not occupationally exposed) of 18.3% for men and 10.1% for women (Magnani *et al*, 1997). An ecological study in Piedmont found that for a unit increase in the relative risk of pleural neoplasm, a 2.5% increase in LC mortality was estimated and 3.9% LC deaths were attributable to residence in municipalities with high pleural neoplasm mortality (proxy of asbestos exposure) (Martuzzi *et al*, 1998). Our study extends this last approach to the whole country and it might be applied wherever registration systems of diseases related to long-term exposure to asbestos are available. Comparing these findings with compensation details from the Italian National Institute for Insurance evidences a relevant gap: in 2000–2004, compensation was given in 2370 asbestos-related mesothelioma cases and to 913 asbestos-related LC cases, with a mean of 474 and 183 cases per year, respectively, configuring an underestimate of around four-fold with respect to the epidemiological findings.

Efforts in developing methods of risk prediction for LCs are strongly encouraged, particularly in Italy, where the markedly reduced consumption of asbestos in some western countries in the 1970s and 1980s did not take place. Early detection measures in asbestos-exposed individuals, unlike in highly lethal mesothelioma, could reduce mortality.

## Figures and Tables

**Table 1:** Annual age standardised rates (per 100 000 inhabitants) for lung cancers by altitude, urbanisation degree, deprivation index and pleural tumours magnitude

**Variable**	**Modality**	**Number of municipalities**	**Annual rates (per 100 000)**	**Test of mean differences and size effect**
Altitude (m)	0–250	3581	95.04	F ANOVA=217.5* P*-value<0.001
	250–500	2297	75.65	
	500+	2223	64.70	
Urbanisation	Urban	862	95.38	F ANOVA=413.9* P*-value<0.001
	Semiurban	2811	96.12	
	Semirural	2257	85.46	
	Rural	2150	65.38	
Deprivation	Very rich	1955	80.76	F ANOVA=15.9* P*-value<0.001
	Rich	1901	89.26	
	Medium	1176	93.34	
	Deprived	1445	88.83	
	Very deprived	1616	93.06	
Pleural tumours mortality rates (per 100 000)	0	5114	77.83	Correlation coefficient Pearson's *r*=0.15* P*-value<0.001
	0–3	1611	90.29	
	3+	1376	99.06	
Overall		8101	89.05	

ANOVA=analysis of variance.

Italy, 1980–2001, males.

**Table 2:** Generalised linear models for lung cancer deaths by altitude, urbanisation degree, deprivation index and pleural cancer mortality risk

**Models**	**Goodness of fit (*R*^2^)**	**Estimated pleural risk coefficient *β*_0_**	**Estimated asbestos-related lung cancer deaths (per year)**	**Percentage of asbestos-related lung cancer deaths (%)**	**Estimated pleura/lung cancer deaths ratio**
(1) ln(O_*i*_/E_*i*_)=*α*+*β*_0_ × PL_*i*_	0.02	2.43	762	3.7	1 : 1.37
(2) ln(O_*i*_/E_*i*_)=*α*+*β*_0_ × PL_*i*_+*β*_1*j*_ × A_*j*_+*β*_2*l*_ × U_*l*_	0.27	1.16	429	1.8	1 : 0.77
(3) ln(O_*i*_/E_*i*_)=*α*+*β*_0_ × PL_*i*_+*β*_1*j*_ × A_*j*_+*β*_2*k*_ × D_*k*_	0.11	1.93	686	3.0	1 : 1.24
(4) ln(O_*i*_/E_*i*_)=*α*+*β*_0_ × PL_*i*_+*β*_1*j*_ × A_*j*_+*β*_2*k*_ × D_*k*_+*β*_3*l*_ × U_*l*_	0.29	0.92	379	1.6	1 : 0.68

A=altitude (factor); D=deprivation index (factor); E=expected lung cancer deaths; O=observed lung cancer deaths; PL=pleural cancers mortality risk (covariate); U=urbanization index (factor).

*β*: regression coefficients; *i*: Italian municipalities=1,…, *N*=8101; *j*=1,…, 3; *k*=1,…, 5; *l*=1,…, 4.

Goodness of fit, estimated asbestos-related lung cancer deaths, estimated pleura/asbestos related lung cancer deaths ratio. Italy, 1980–2001, males.
